# Response to the letter by Prof Jonathan Deeks to the Lancet EClinicalMedicine editor

**DOI:** 10.1016/j.eclinm.2021.101104

**Published:** 2021-09-11

**Authors:** Werner Leber, Oliver Lammel, Jasmina Panovska-Griffiths, Thomas Czypionka

**Affiliations:** a Wolfson Institute of Population Health, Queen Mary University of London, UK; b Practice Doc Lammel, Ramsau am Dachstein, Austria; c Big Data Institute, Li Ka Shing Centre for Health Information and Discovery, Nuffield Department for Medicine, University of Oxford, Oxford, UK; d Institute for Advanced Studies, Vienna, Austria

We thank Prof Deeks for taking the time to read our study and respond to our published manuscript [1]. He argues that “The high sensitivity [of the study] is created by a flaw in the study design, as has the low specificity”; that “Incomplete verification linked to the result of the LFT” has led to the sensitivity being overestimated and the specificity being underestimated; and that the 1525 patients with “negative” LFT were “intentionally excluded from verification”.

We disagree that we have intentionally excluded the 1525 patients. Instead, we have clearly articulated in the manuscript that we excluded 1525 patients from the analysis because these patients were deemed not suspected of COVID-19 on the basis of both clinical assessment and non-reactive LFT result. This was according to the national protocol of the recommended use of LFT in symptomatic patients across primary care in Austria.

Specifically, on 22nd October 2021, Austria published guidelines recommending offer of LFT to symptomatic patients attending healthcare settings, including primary care. Our paper (REAP2) evaluates the pragmatic implementation of these guidelines across a large primary care network of 20 general practices in the district of Liezen [1]. The network was set up on recommendation of the initial study (REAP1) evaluating RT-PCR testing in a single general practice [2] and in preparation for a looming second COVID-19 wave in the autumn 2020. Payments for LFT testing to practices by sickness funds are performance-linked and are stratified in three groups according to COVID-19 risk using a combination of pre-test probability in clinical triage and LFT result as per the following categories:

COVT1: High risk suspected COVID-19 AND reactive LFT,

COVT2: Not at risk of COVID-19 AND non-reactive LFT,

COVT3: Moderate risk suspected COVID-19/uncertain diagnosis AND non-reactive LFT.

According to the national protocol, confirmatory RT-PCR testing was required for COVT1 (referred to as Group 1 in REAP2) and COVT3 (Group 2 in REAP2), but not for COVT2. The 1525 patients excluded from the study are equivalent to COVT2 and not suspected of COVID-19 due to clinical triage using a combination of clinical assessment and non-reactive LFT. Therefore, under the national protocol, they were not RT-PCR tested. Hence, we re-iterate that the exclusion of 1525 patients was on clinical grounds as per the national protocol, but not “intentionally” to skew validation of data as Prof Deeks suggests. We note that the aim of the work in REAP2 was to evaluate the accuracy of LFT in suspected patients for whom RT-PCR was available and this included the 1027 patients (40% of the total patients) studied.

Prof Deeks suggested that all 2562 patients, including 1027 suspected cases (826 RT-PCR positive, 201 RT-PCR negative, 10 no RT-PCR data) and 1525 not suspected cases, should have been included in the analysis. Given the lack of RT-PCR data for the latter, he suggested that, alternatively, the analysis should have been adjusted by multiplying the LFT “negatives” by a factor of “eight” (1525/217 = 7.0). However, doing this would have “overcounted” the number of negatives as it would have included patients unlikely to have had COVID-19 (e.g., those presenting with food poisoning, glandular fever, or bacterial tonsillitis, etc.). Given the combination of clinical assessment and non-reactive LFT, patients in COVT2 can be assumed to have very low pre-test probability when compared to moderate (COVT2) and high risk (COVT1) suspected cases.

To estimate the percentage of patients not suspected of COVID-19 prior to LFT testing that may have tested RT-PCR positive if all 1525 patients were RT-PCR tested, we emailed all 20 participating general practitioners (GPs) asking if any of the 1525 patients should have been included. 15/20 GPs replied suggesting that a range of 1–10% had the potential to be included if they were RT-PCR tested. Specifically, 4 practices suggested 1% of patients, 6 practices suggested between 3–5%, 3 < 5% and 2 between 5 and 10%. Based on this, although aware of a risk of potential recall bias, we would consider these estimates as fairly accurate as all practices are single-handed fostering a culture of close relationship between patients and their doctor. Using this data, we undertook a sensitivity analysis on the accuracy adjusting for 5 and 10% reclassification i.e., addition to the pool of LFT non-reactive from the pool of 1525 patients (see Table 1). With these adjustments, sensitivity decreased slightly while specificity increased slightly. Namely, assuming 5% reclassification, estimated sensitivity was 93.9% and specificity was 91.7% while assuming 10% reclassification, sensitivity was 92.4% and specificity 93.3%. We note that these estimates are only marginally different to the sensitivity (95.4%) and specificity (89.1%) reported in the published REAP2 paper. Clinical accuracy of a test varies according to disease prevalence. Sensitivity of a test increases with increased prevalence, whilst its specificity decreases [3]. Therefore, the high sensitivity and low specificity observed in our study likely are a function of high disease prevalence (positivity rate 79.7%) among suspected cases undergoing clinical triage using both clinical assessment and clinician-led testing during a major outbreak. In addition, the relatively low specificity may represent faulty test kits, device storage issues and difficulty in test interpretation when using five different LFT products across 20 practices reporting initial data from the first 2 months of implementation.

**Table 1:** Unadjusted accuracy (defined by sensitivity, specificity, positive predictive value and negative predictive value) of lateral flow testing (LFT) when compared to reverse transcriptase-polymerase chain reaction (RT-PCR) on data from 1027 patients from a general practice network in Austria.

	RT-PCR negative	RT-PCR positive	Total tests	
LFT reactive	22	788	810	Positive Predictive Value 97.3%
LFT non-reactive	179	38	217	Negative Predictive Value 82.5%
Total tests	201	826	1027	
	Specificity 89.1%	Sensitivity 95.4%		
b. Estimated adjusted accuracy of LFT assuming **5%** of patients clinically not suspected of COVID-19 that may have tested RT-PCR positive without LFT testing. This means we need to redistribute the 76 (0.05×1525) patients into LFT non-reactive using the same ratio as before for RT-PCR positive and negative. As a result, sensitivity drops slightly, and specificity increases slightly.				
	RT-PCR negative	RT-PCR positive	Total tests	
LFT reactive	22	788	810	Positive Predictive Value 97.3%
LFT non-reactive	242 (= 179/217 × 293)	51 (= 38/217 × 293)	293 (= 217 + 76)	Negative Predictive Value 82.6%
Total tests	264	839	1103 (1027 + 76)	
	Specificity 91.7%	Sensitivity 93.9%		
c. Estimated adjusted accuracy of LFT assuming **10%** of patients clinically not suspected of COVID-19 that may have tested RT-PCR positive without LFT testing. This means we need to redistribute the 153 (0.1 × 1525) patients into LFT non-reactive using the same ratio as before for RT-PCR positive and negative. As a result, sensitivity drops slightly, and specificity increases slightly.				
	RT-PCR negative	RT-PCR positive	Total tests	
LFT reactive	22	788	810	Positive Predictive Value 97.3%
LFT non-reactive	305 (= 179/217 × 370)	65 (= 38/217 × 370)	370 (= 217 + 153)	Negative Predictive Value 82.4%
Total tests	327	853	1180 (1027 + 153)	
	Specificity 93.3%	Sensitivity 92.4%		

We would also like to use this opportunity to clarify three aspects around LFT testing. Firstly, we note that we referred to LFT results as “reactive” and “non-reactive” rather than “positive” and “negative” to account for the “preliminary” or “presumptive” nature of LFT until confirmed by RT-PCR. Secondly, performance of LFT widely varies between healthcare and community settings, the mode of testing (clinician-led vs. self-testing), and the health status of the individual tested (asymptomatic/presymptomatic vs. symptomatic). Thirdly, we agree with Prof Deeks that testing the patients not suspected with COVID-19 with an RT-PCR test could have been an alternative way to analyze the data. However, given the much lower pre-test probability among this group, we would have analyzed this data separately from the cohort of 1027 COVID-19 suspected cases.

## Declaration of Competing Interest

None declared.
